# Sodium Butyrate Inhibits Neovascularization Partially via TNXIP/VEGFR2 Pathway

**DOI:** 10.1155/2020/6415671

**Published:** 2020-11-20

**Authors:** Xiaoqiang Xiao, Min Chen, Yanxuan Xu, Shaofen Huang, Jiajian Liang, Yingjie Cao, Haoyu Chen

**Affiliations:** Joint Shantou International Eye Center, Shantou University and the Chinese University of Hong Kong, Shantou, China

## Abstract

Control of neovascularization with small molecules is a promising tactics. Here, we tested the roles of sodium butyrate (NaBu) on the neovascularization and primary explained its underlining molecular links. We used models including cell and ex vivo culture of choroid and mouse, as well as the biochemical and cellular techniques, to confirm our hypothesis. We found that treating HUVEC cells with NaBu (both 2.5 mM and 5 mM) significantly inhibited its ability in tube formation and proliferation. This inhibitory effect was also observed in choroid sprouting experiments, compared to the control. Interestingly, the choroid sprouting suppressed by NaBu can proliferate again after removing it, indicating that the cell cycle progression might be arrested. The laser-induced choroid neovascularization (CNV) was significantly alleviated by assessing the CNV size (decreased to 0.73 fold) in contrast with the vehicle control group after 2.5 mM NaBu injection for 7 days. Mechanistically, we found an enhanced TXNIP expression in response to NaBu treatment in all the three models. Overexpressing TXNIP in HUVEC cells blocked its tube formation and inhibited its proliferation; on the other hand, knocking down its expression with shRNA reversed those phenotypes in context of NaBu treatment. Further investigation showed the expression of VEGF receptor 2 (VEGFR2) in HUVEC cells was regulated by TXNIP undergoing NaBu treatment. We therefore argued that NaBu inhibited neovascularization partially through TXNIP-regulated VEGFR2 signal pathway.

## 1. Introduction

Neovascularization is a common change in multiple ocular diseases, including retinopathy of prematurity, diabetic retinopathy, and neovascular age-related macular degeneration [1]. The neovessels show defect in tight junction and damage to vessel stability, leading to pathogenesis of various diseases [1]. Accumulated evidences show vascular endothelial cell growth factors (VEGFs), and their receptors (VEGFRs) establish complex pathways to delicately regulate vessel formation in angiogenesis [2]. As a main VEGF-binding receptor, VEGFR2 (also known as Flk-1 or KDR) can activate various signaling cascades in response to VEGF stimuli [2, 3]. Modifications of VEGFR2 protein with phosphorylation, ubiquitylation, and SUMOylation are crucial to its signaling cascades [4]. For instance, SUMOylation at Lys1270 on VEGFR2 induces its accumulation at the Golgi, blocking VEGFR2 trafficking to cell surface and signaling [3].

The development of anti-VEGF therapies, such as pegaptanib, ranibizumab, bevacizumab, and aflibercept, had major advances in controlling the angiogenesis and vascular permeability [5]. However, the frequent injection of those medicines highly enhanced the physical and economic burden of patients. Moreover, the current therapeutic methods including anti-VEGF therapies show several other side effects such as individual specificity [6–9]. Therefore, it is urgent to develop novel methods, which are effective, cheap, and low individual specificity [10, 11].

Butyrate is one of short chain fatty acids (SCFAs) produced by intestinal bacteria in gut which ferment food fiber. As an endogenic histone deacetylase (HDAC) inhibitor, butyrate remodels the acetylating state on histone and thus gene expression profile [12, 13]. It was also reported that butyrate increases the activity of histone acetyltransferases (HAT) and promotes the destined gene transcription [12, 14, 15]. In light of its profound effect on gene expression, butyrate affects various cellular events, including proliferation, cell cycle, apoptosis, differentiation, migration, and invasiveness [12, 16–19]. Butyrate was confirmed to be an effective small molecule in inhibition of diabetes and various tumors [13, 20, 21].

Our previous study showed butyrate-induced cell death and cell cycle arrest on non-small-cell lung cancer line A549 partially via thioredoxin interacting protein (TXNIP), which expression was highly induced by the treatment of NaBu [22]. TXNIP binding to the thioredoxin (TRX) plays key roles in regulating cellular redox homeostasis [9, 23–25]. TXNIP also activates inflammasome NLRP3 and participates in the inflammatory response [16, 26–28]. Apart from above stated functions, TXNIP also controls glucose transporter 1 (GLUT1) recycle on cellular membrane and leads to the block of glucose uptake and metabolism [29].

Here, we investigated the roles of NaBu on neovascularization and dissected its molecular mechanisms.

## 2. Materials and Methods

### 2.1. Animals

Animals used in this study were adhered to the ARVO Statement for the Use of Animals in Ophthalmic and Vision Research and were approved by the animal ethics committee of Joint Shantou International Eye Center. C57BL/6J mice used in this study were purchased from Vitalriver (Charles River Laboratories, Beijing, China). The mice were placed in SPF environment, treated with 12 hours light and 12 hours darkness with free access to food and water. In order to investigate the effect of NaBu to the neovascularization in vivo, the mouse was always used to these kinds of analysis. We totally used about 100 mice in our current research and selected one eye for treatment, and the other eye was used for control.

### 2.2. Endothelial Cell Culture and Transfection

HUVEC (human umbilical vein endothelial cells) used in this study were kindly gifted by the department of pathophysiology of Shantou University Medical College. The cells were cultured in Dulbecco's Modified Eagle's Medium (DMEM; Gibco) supplemented with 10% fetal bovine serum and 1% penicillin–streptomycin. All cells were maintained in a humidified atmosphere of 5% CO2 at 37°C. For overexpression, the TXNIP sequence (Primers: FP, ggatctatttccggtgaattcgccacc ATGGTGATGTTCAAGAAG; RP, agaactagtctcgaggaattc CTGCACATTGTTGTTGA) was cloned into pHB-EF1-MCS-GFP vector. TXNIP shRNA expression vectors were as the same as our previous paper [11]. A nonsense scrambled shRNA sequence was used as a negative control. The TXNIP shRNA expression or negative control plasmids were transfected into HUVEC cells together with two lenti-virus package plasmids using LipoFiter according to manufacturer's instructions (LipoFiter TM Liposomal Transfection Reagen, HangHeng biology). The medium was changed 24 hours posttransfection, and puromycin (1 *μ*g/ml) was used to screen for stable cell line. The TXNIP expression and its corresponding empty vector were transfected into the designated cell lines. TXNIP expression level was verified by western blot or qPCR.

### 2.3. *In vitro* Cell Proliferation Assay (MTT)

Cell proliferation assay was performed according to previous protocol [30]. Briefly, HUVEC cells were seeded in 96-well plate (9 × 10^3^ cells/well) and incubated at 37°C overnight. The medium was then replaced with fresh medium added with vehicle or different dosage of NaBu (1, 2.5, and 5 mM) and incubated for 24, 48, and 72 h, respectively. Cell proliferation was determined with 5 mg/ml MTT (Thermo, USA) incubation for 3 h at 37°C; the MTT solution was removed, and 100 *μ*l isopropanol was added to dissolve the precipitate. The absorbance was measured under 570 nm wavelength in spectrophotometer (ND-2000, Thermo, USA). Experiments were performed at least three times with six repeats per sample for each time.

### 2.4. *In Vitro* Tube Formation Assay

The capillary-like tube formation was performed on 3D Matrigel Matrix (BD Biosciences, 356230) according previous protocol [31]. Briefly, Matrigel was 1 : 1 diluted with EBM-2 at 4°C and precoat 48-well plate with 200 *μ*l/well. After polymerizing for 30 min at 37°C, HUVEC cells (5 × 10^4^/well) resuspended in DMEM medium with or without the presence of NaBu (1, 2.5, and 5 mM) were then added into the above plates. After 24 h incubation at 37°C, the tubule-like structure comes the best condition; 8 fields were randomly selected in each well under an inverted microscope (Nikon, USA). The tubulin-like structure was quantified by measuring the total tubule length, size, and junction by an automated image analysis tool, MATLABR-based program AngioQuant (The MathWorks, Natick, MA, USA) and expressed as % of the control group. The experiments were repeated at least three times.

### 2.5. Western Blot Analysis

Western blot was performed according to previous protocol [32]. Briefly, cells or tissues were collected and used for protein extraction. The lysated proteins from different treatments were applied (50 *μ*g) to SDS-PAGE and then transferred onto PVDF membranes (Millipore, USA). After blocked with 5% milk for 1 hour at room temperature, the membranes were incubated at 4°C overnight with primary antibodies; after washed for 3 times, secondary antibodies were incubated for 2 hours at room temperature. The protein stripes were visualized by chemiluminescence with AB (cat. no. NCI4106. Thermo, USA) incubation for 5 minutes and captured on film. The bands' density was analyzed with the ImageJ software (National Institutes of Health). The primary antibodies are used here including anti-TXNIP (cat. no. ab188865; Abcam), anti-VEGFR2 (cat. no. 2479s; Cell Signaling), and anti-*β*-actin (cat. no. KC-5A08; Shanghai kangcheng biotechnology). The secondary antibodies were sheep anti-rabbit IgG antibody (cat. no. #7O74S; Cell Signaling).

### 2.6. Choroid Sprouting Assay

The choroid sprouting assay was performed according to previous protocol [32, 33]. Briefly, choroid tissues (retinal pigment epithelium/choroid/sclera complex) from P8 (postnatal 8 days) C57BL/6J mice was dissected on ice and cut into approximately 1mm × 1mm pieces. The pieces were then placed in 24-well plates precoated with growth factor-reduced Matrigel (cat. no. 356230; BD Bioscience, San Jose, CA, USA) and incubated at 37°C. After 10 minutes, the Matrigel was solidified and 500 *μ*l EGM-2 medium (cat. no. cc-3156; Lonza, CA, USA) with growth factors kit (cat. no. cc-4176; Lonza) was added into each well. Different concentrations of NaBu (0, 0.1, 0.5, 1.0, 2.5, and 5.0 mM) were then added to the medium at designated time points. The medium was changed every 3 days, and choroid sprouting was observed each day with an inverted microscope. The choroid sprouting ability was quantified by measuring the pixel number of the sprouting microvascular using threshold tool of ImageJ. The tolerance was set at 30.0, through adjusting the threshold value; the area of sprouting microvasculature was measured in pixel number. The results were expressed as fold-change from day 2 to day 4 or day 6 [33, 34].

### 2.7. Laser-Induced Choroid Neovascularization and Vascular Leakage

Choroid neovascularization was induced by laser photocoagulation of Bruch's membrane with the Coyne laser machine (NOVUS, Spectra, Japan). Briefly, C57BL/6J mice (2-3 months postnatal) were subjected to pupil dilation with tropicamide (Alcon, Fort Worth, TX, USA) and anesthetized with ketamine/xylazine. Laser photocoagulation (120 mW, 20 ms, 75 *μ*m spot size) was performed with four laser burns in the 3, 6, 9, and 12 o'clock position of the posterior pole of the fundus with the distance of 2-3 disc diameters from the optic nerve head. Only burns with bubble formation indicating breakage of Bruch's membrane and this is the successful CNV model in the study. NaBu (1, 2.5, and 5 mM) was intravitreally injected at day 0 and day 3 after photocoagulation. 6 days after laser photocoagulation, mice were anesthetized and pupils dilated and subjected to fundus fluorescein angiography (FFA). Briefly, 20 mg/ml sodium fluorescein (Guangzhou Baiyunshan Mingxing Pharmaceutical Co. LTD) was diluted with 0.9% NaCl into 5 mg/ml and injected intraperitoneally at 0.01 ml/10 g body weight. Fluorescent fundus images with CNV leakage in the posterior fundus were taken with ROLAND Consult (Germany) system at 4-6 minutes after injection. At 7 days after laser photocoagulation, CNV reached its peak size; the mice were anesthetized and euthanized by cervical dislocation. The eyes were immediately enucleated and fixed in 4% paraformaldehyde (PFA) in PBS for 2 hours at room temperature. The posterior eye cups consisting of the retinal pigment epithelium (RPE)/choroid/sclera were then dissected, and the choroid NV tufts were labeled with Isolectin IB4 (cat. no. L2895; Sigma-Aldrich, Saint Louis, Missouri 63103 USA) in 0.2% Triton-X100 and 0.5 mM MgCl_2_ in PBS overnight. After washed with PBS for 3 times and 5 minutes each, the posterior eye cups were cutted into 4 parts like blossom shape with optic nerve head connected and mounted onto slides with scleral side down. Fluorescent images were taken at 10x and 20x magnifications under a laser scanning cofocal microscope (Leica Microsystems) [35].

### 2.8. Quantification of Choroid Neovascularization and Vascular Leakage

The quantification of choroid neovascularization and vascular leakage was both performed with the ImageJ (National Institutes of Health) software. Briefly, the “area” function of ImageJ was selected and used to quantify the CNV area labeled with Isolectin B4; the results “square inch” were converted into “square micron” for quantification. The “integrated intensity” function of ImageJ was selected and used to quantify the fluorescence intensity of CNV leakage. The results were quantified and recorded as pixel number. To eliminate errors, burns with bleeding, fused lesions, and lesions five times more than the mean lesion size in the same group were excluded from the study [35]. The quantification was performed by at least two independent masked observers to the identity of samples.

### 2.9. Immunofluorescence Histochemistry

The laser-induced whole-mount choroids stained with Isolectin IB4 were further subjected to immunofluorescence histochemistry (IHC). Briefly, the RPE/choroid/sclera complex was subjected to 0.3% Triton X-100 for permeabilization and 5% anti-goat serum in PBS to block the antigen for 1 hour. The tissues were then incubated in primary antibody dilution of TXNIP (1 : 500, cat. no. ab188865; Abcam, Cambridge, MA, USA) with 0.3% Triton X-100, 2% NDS in PBS overnight at 4°C. After washed for 4 times and 5 minutes each, the tissues were incubated with corresponding secondary antibody (Alexa Fluor Plus 555 Goat anti Rabbit IgG (H+L), A32732; Invitrogen, USA) for 2 hours at room temperature, followed by mounting onto slides. Images were taken at 20x magnification under a laser scanning confocal microscope (Leica Microsystems).

### 2.10. RNA Extraction, Reverse Transcription, and RT- PCR

Cells or tissues were harvested with Trizol reagent (cat. no. 15596026, Thermo Scientific, USA). The samples were homogenized with pestles, followed by RNA extraction using Trizol method according to the manufacturer's instructions. The extracted RNA was then reverse transcripted into cDNA using a Reverse Transcription kit (cat. no. # RR037A; TaKaRa, Japan). Then, the designated cDNA was set as a template, and RT-PCRs were carried out using gene-specific primer sets (cat. no. # RR420A TaKaRa, Japan). Gene expression was calculated by relative to housekeeping control genes using 2^-*ΔΔ*CT^ method. Polymerase chain reaction primers for the housekeeping control genes ACTB and target genes were designed using the NCBI Primer Blast database (http://www.ncbi.nlm.nih.gov/tools/primer-blast/) Table 1.

### 2.11. Statistical Analysis

Data are presented as mean ± SEM. Student's *t*-test was used to compare between 2 groups of samples. For multiple comparisons, two-way ANOVA followed by Tukey's post hoc test was performed using Prism 6 (Graph-Pad, San Diego, CA). The criterion for significance was set at a probability of *P* ≤ 0.05.

## 3. Results

### 3.1. NaBu Treatment Inhibits HUVEC Cells Proliferation and Tube Formation

**Figure 1:**
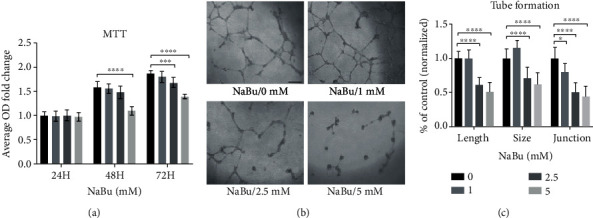
NaBu treatment inhibits HUVEC cells proliferation and tube formation. (a) HUVEC proliferation analysis was performed with HUVEC cells, which were seeded on 96-well plate (9000 cells/well) with (0 mM) or without NaBu (1, 2.5, and 5 mM) treatment for 24, 48, and 72 hours. Cell proliferation was determined with 5 mg/ml MTT (Thermo Scientific, USA) incubation for 3 hours, followed by dissolving in isopropanol. The absorbance measured under 570 wavelength showed the inhibited proliferation ability under NaBu treatment in a dose and time dependent manner (*n* = 6‐8, ^∗∗∗^*P* ≤ 0.001, ^∗∗∗∗^*P* ≤ 0.0001). (b) HUVEC cells were digested and suspended in EBM-2 medium containing different dosage of NaBu (0, 1, 2.5, and 5 mM); then, the prepared cells (5 × 10^4^/well) were seeded onto 48-well plate precoated with Matrigel. After 24 hours incubation, images were obtained by an inverted microscope (*n* = 6‐8, bar: 400 *μ*m). (c) Quantification of tube formation with the ImageJ software: total tubule length, size, and junction (*n* = 6‐8, ^∗^*P* ≤ 0.05, ^∗∗∗∗^*P* ≤ 0.0001).

NaBu can arrest cancer cell proliferation and growth [11]; we therefore tested whether it also blocked the proliferation of human umbilical vein endothelial cells (HUVEC). We then treated the cells with different concentration of NaBu (0, 1, 2.5, and 5 mM) and incubated for different time points (24, 48, and 72 h). After NaBu treated, cells survival rates were assessed by MTT. As showed in Figure 1(a), NaBu significantly inhibited the proliferation of HUVEC cells at 5 mM, 48 hours, and 2.5 mM at 72 hours, respectively. Next, we examined the effect of NaBu on the initial phase of angiogenesis and vascularization via HUVEC cell tube formation assay. To observe this, we seeded 5 × 10^4^ cells/well, which were pretreated with NaBu (0, 1, 2.5, and 5 mM), on Matrigel. The seeded cells were further treated with the corresponding concentration of NaBu for a period of designated time points. After continue observation of the tube formation progress, we took picture and calculated the total tubule length, size, and junction of tube formation with the ImageJ software. As expected, NaBu inhibited the HUVEC tube formation in a dose-dependent manner (Figure 1(b)). 5 mM NaBu could greatly block the formation of HUVEC tube (Figure 1(b)). Statistical analysis showed total tubule length significantly decreased at 2.5 mM (0.61 folds) and 5 mM (0.51 folds), comparing to the vehicle treated control (Figure 1(b)). In consistent with the results of tubule length, the hole size, and the number of junction also significantly reduced, size (2.5 mM (0.71 folds) and 5 mM (0.62 folds)) and junction (1 mM (0.80 folds), 2.5 mM (0.50 folds), and 5 mM (0.44 folds)), respectively, comparing to the vehicle control (Figure 1(c)). Those data strongly indicate NaBu might inhibit neovascularization.

### 3.2. NaBu Treatment Blocked Choroid Sprouting and Laser-Induced Choroidal Neovascularization

**Figure 2:**
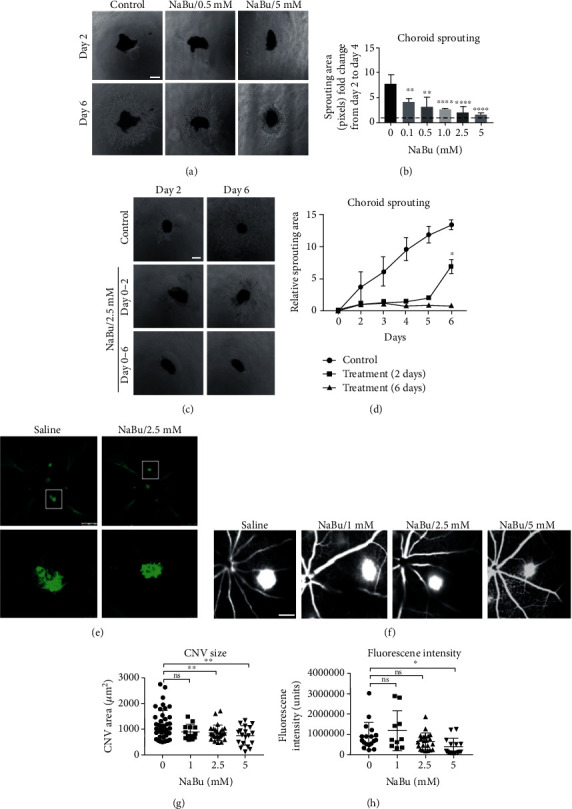
NaBu treatment inhibits choroid sprouting and laser-induced neovascularization. (a) Choroid sprouting assay was performed by dissecting the choroid from ocular of P8 mice and cultured in EGM-2 medium for 2 days. Then, NaBu with designated concentrations was added and incubated for another 2 days. Images were taken under an inverted microscope at the designated time points. (b) Quantification of the sprouting area was calculated as fold-change of pixels units by ImageJ from day 2 (dashed line) to day 4 and normalized to the control group. There was a significant inhibition on the sprouting area from 0.1 mM NaBu and the effect strengthened with increased dosage (*n* = 4‐6, ^∗∗^*P* ≤ 0.01, ^∗∗∗^*P* ≤ 0.001, ^∗∗∗∗^*P* ≤ 0.0001, bar: 400 *μ*m). (c) Continuous treatment from day 0 to day 6 (lower panel), treated for 2 days and then removed NaBu (middle panel), without NaBu-treated group (upper panel). (d) The growth trends were quantified as pixel number in each group by ImageJ (*n* = 4‐6, ^∗^*P* ≤ 0.05, bar: 400 *μ*m). (e) Wild-type C57BL/6J mice subjected to laser-induced choroid neovascularization were intravitreously injected NaBu (2.5 mM) at day 0 and day 3 (two times) or vehicle (saline). Seven days later, choroid tissues were dissected and stained with Isolectin IB4 (green). Images were taken under a cofocal laser scanning microscope. (f) At 6 days after photocoagulation, CNV leakage was imaged under ROLAND consult at 4-6 minutes after fluorescence sodium injection. (g) Quantification of CNV lesion sizes showed that 2.5 mM NaBu treatment effectively inhibited choroid neovascularization and the effect increased with dose-dependent manner (*n* = 13‐26, ^∗∗^*P* ≤ 0.01, bar: 500 *μ*m). (h) The fluorescent intensity was quantified by ImageJ and showed decreased leakage density at 5.0 mM NaBu treatment comparing to the control group (*n* = 13‐26, ^∗^*P* ≤ 0.05, bar: 500 *μ*m).

To further investigate the suppressive effect of NaBu on neovascularization, we used *in vitro* cultured choroid sprouting assay. We took out the mouse choroid tissues (C57, postnatal 8 days) and cut into 1 × 1mm^2^ pieces. The choroid pieces were then embedded into Matrigel and cultured with EGM2 medium. After two days culture, the medium supplemented with different concentrations of NaBu was added into the cells and continued to culture for the designated time points. The results showed that NaBu strongly inhibited the ability of choroid sprouting even at 0.1 mM concentration (Figures 2(a) and 2(b)). Quantifying the sprouting with ImageJ was performed, and the results showed a dosage-dependent suppressive effect on choroid sprouting after six days continued treatment. The fold changes were as follows: 0.52 fold for 0.1 mM, 0.41 fold for 0.5 mM, 0.34 fold for 2.5 mM, and 0.20 fold for 5 mM, respectively, comparing to vehicle control (Figure 2(b)). We further examined the ability of choroid sprouting after removing the NaBu treatment. Interestingly, the choroid vessels resprouted after the removal of NaBu, more than 9 folds increment comparing to the continued treatment controls (Figures 2(c) and 2(d)). This result indicates NaBu-mediated sprouting suppression might interfere with cell cycle progression rather than induce cell death. Laser-induced mouse choroid neovascularization model was also used to assess the *in vivo* suppressive roles of NaBu on neovascularization. Mice (two months age) were intravitreously injected different dosages of NaBu (1, 2.5, and 5 mM) or PBS controls (pH = 7.4) for two times at day 0 and day 3, respectively, after laser induction. At day six, the above mice were injected with fluorescence sodium after anaesthetization, and the vascular leakage of CNV lesions was analyzed. Fluorescein intensity of CNV lesions was then calculated as follows: 406052 ± 103781 units for 5 mM NaBu and 919013 ± 156664 units for control (*P* value ≤ 0.05; Figures 2(f) and 2(h)), with ImageJ. Seven days later, mice choroids were euthanized, and the whole mount choroids stained with Isolectin B4 were performed. The neovascularization (CNV) regions on choroids were photographed by a cofocus microscope (Figure 2(e)) and calculated by the ImageJ software. Statistically, CNV lesion sizes were 857.8 ± 57.46 *μ*m^2^ for 2.5 mM, 760.5 ± 84.41 *μ*m^2^ for 5 mM NaBu, and 1175 ± 81.41 *μ*m^2^ for PBS control group (Figures 2(e) and 2(g)). After analyzing their *P* values, we found the concentrations of both 2.5 and 5 mM for NaBu showed significant reduction in neovascularization in contrast with the control (Figures 2(e) and 2(g)). Those data obviously showed NaBu powerfully suppressed CNV in vivo.

### 3.3. NaBu Highly Induced TXNIP Expression *In Vivo* and *In Vitro*

**Figure 3:**
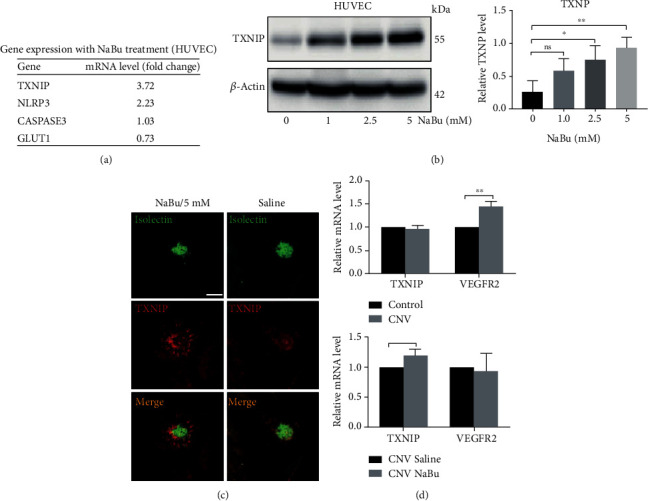
TXNIP expression was highly induced by NaBu *in vitro* and *in vivo.* (a) qPCR was performed to detect the mRNA expression of TXNIP, NLRP3, caspase 3, and GLUT1. HUVEC cells were treated with 2.5 mM NaBu for 24 hours and then used for total RNA extraction with Trizol. After the synthesis of cDNA, realtime-PCRs (qPCR) were performed with corresponding primer sets. Among those four genes, TXNIP was highly induced (~4 folds). (b) HUVEC cells were treated with designated concentration of NaBu for 48 hours and then collected for western blot analysis with anti-TXNIP antibody (^∗^*P* ≤ 0.05, ^∗∗^*P* ≤ 0.01). (c) Laser-induced choroid neovascularization stained with FITC-Isolectin IB4 was subjected to immunohistochemistry staining of TXNIP. Compared to control group, the CNV treated with NaBu has a higher TXNIP expression which costained with Isolectin IB4 but much more intense in the peripheral area of CNV region (bar: 400 *μ*m). (d) Laser-induced CNV membrane was collected for RNA extraction and used to qPCR analysis. Laser photocoagulation highly induced the VEGFR2 expression (^∗∗^*P* ≤ 0.01).

NaBu induction remodels the cellular genes expression profile. Previously, we found TXNIP could be highly induced by NaBu in A549 cells. Here, we further investigated the TXNIP expression in HUVEC cells and mouse choroid in response to NaBu treatment. As expected, comparing to the vehicle controls, both the mRNA and its protein of TXNIP showed higher level expression in the NaBu-treated group, and the enhanced expression was in a dosage-dependent manner (Figures 3(a)–3(c)). In HUVEC cells, the expression of TXNIP increased 3.72 folds in mRNA (Figure 3(a)) and more than 2 folds in protein (Figure 3(b)), respectively. The enhanced TXNIP expression was further validated in choroid sprouting via immune-staining with TXNIP antibody (Supplemental Figure 1). The expressions of NLRP3, caspase 3, GLUT1, and VEGFR2, which are involved in either inflammation, apoptosis, glucose transportation, or angiogenesis, were also tested. The mRNA expressions of NLRP3 and GLUT1 showed slight change: Nlrp3 increased 1.27 folds, and GLUT1 decreased 0.72 folds, respectively, in contrast to the control (Figure 3(a)). Interestingly, the VEGFR2 expression significantly increased in response to 2.5 mM NaBu treatment in HUVEC cells (up to 5.96 folds) (Figure 3(a)). After NaBu treatment, an enhanced protein TXNIP signal was also observed at or around the laser-induced CNV lesions, which was stained by Isolectin IB4, in comparison with the controls (Figure 3(c)). And this upregulation was also observed in mRNA (Figure 3(d), low panel). Furthermore, we found that the TXNIP expression could not be induced in response to the laser stimulation (Figure 3(d), up panel), but the mRNA expression of VEGFR2 was significantly upregulated in the context of laser induction (Figure 3(d), up panel). However, there was no observable change after further NaBu treatment (Figures 3(c) and 3(d)). Whether NaBu induced apoptosis in other normal ocular tissues, therefore, we did the TUNNEL analysis on the retina and choroid. We found that there was no significant difference between NaBu-treated tissues and control tissues (Supplemental Figure 2). Those results indicate NaBu-mediated inhibition of neovascularization causes little side effect on the normal tissues in our used concentrations.

### 3.4. TXNIP Overexpression Inhibits the Proliferation and Tube Formation of HUVEC Cells

**Figure 4:**
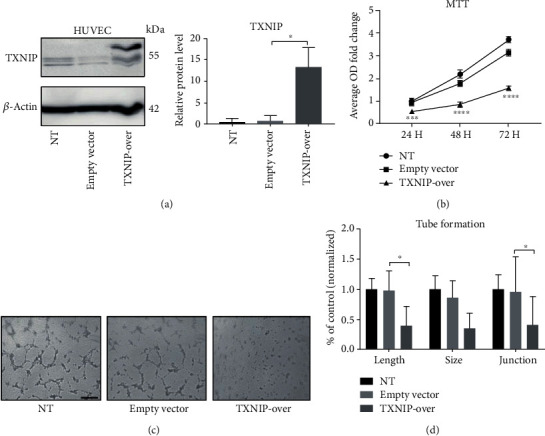
TXNIP overexpression inhibits the proliferation and tube formation of HUVEC. (a) Plasmids containing TXNIP ORF sequence were transfected into HUVEC cells with lipo-filter according to the manufacturer's instructions. Western blot was used to confirmed TXNIP expression (left); relative grey values to beta-actin were used to statistical analysis (^∗^*P* ≤ 0.05) (right). (b) MTT assay was performed with HUVEC cell types in (a). Cells seeded on 96-well plate (9 × 10^3^ cells/well) and incubated for 24, 48, and 72 hours. The medium was changed with MTT for 3 hours, and the OD values at 570 wavelength were measured (*n* = 6‐8, ^∗∗∗^*P* ≤ 0.001, ^∗∗∗∗^*P* ≤ 0.0001). (c) Tube formation assay was performed with HUVEC cells prepared as in (a) and then seeded onto 48-well plates (5 × 10^4^ cells/well) precoated with Matrigel. After 24 hours incubation, the tubule-like pictures were taken under an inverted microscope (bar: 100 *μ*m). (d) Quantification of HUVEC tube formation with ImageJ showed remarkably reduced total tubule vascular length and junction in TXNIP overexpression (*n* = 8, TXNIP-OV) HUVEC cells compared to the empty vector control (E.V) (^∗^*P* ≤ 0.05). TXNIP-OV: HUVEC cells transfected with TXNIP expression vector; E.V: HUVEC cells transfected with corresponding empty vector; NT: HUVEC cells without transfection.

Above data showed TXNIP might participate in regulating NaBu-mediated CNV suppression. We next overexpressed TXNIP protein in HUVEC cells and tested its effect on the proliferation and tube formation of HUVEC cells. The lentivirus plasmid (TXNIP-OV) highly expressed TXNIP protein compared with the empty vector control (E.V) and no transfection group (NT) (Figures 4(a) and 4(b)), and the infection efficiency was marked by the GFP fluorescence (Supplemental Figure 3). Using HUVEC cells infected with TXNIP-OV or empty vector, we found the cells expressing TXNIP showed a significant decrease in tube formation in contrast with the cells expressing empty vector as image showed in Figure 4(c). ImageJ analysis showed the total tube formation length, size, and junction of HUVEC cells expressing TXNIP (TXNIP-OV) were all significantly decreased compared to the cells expressing empty vector (E.V) and no transfection (NT) (Figure 4(d)). We further tested the proliferation ability on HUVEC cells with or without TXNIP overexpression via MTT. We assessed the proliferation ability of those cells at different time points (24, 48, and 72 hours). As in Figure 4(b) showed the proliferation of HUVEC cells transfected with plasmid expressing TXNIP protein significantly reduced in contrast with the cells transfected with empty vectors in all three time points. Those data further showed TXNIP affected the early stage of neovascularization.

### 3.5. Knocking down TXNIP Expression with shRNA Rescued the Proliferation and Tube Formation of HUVEC and Enhanced VEGFR2 Expression in Context of NaBu Treatment

**Figure 5:**
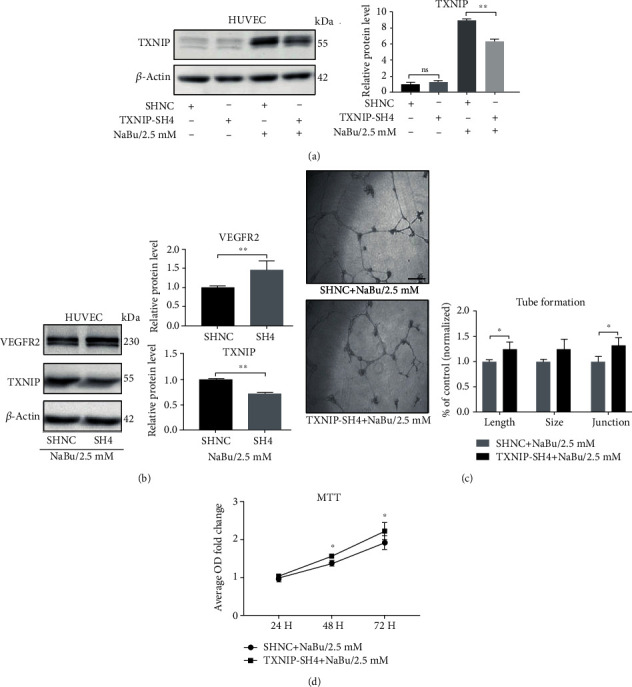
TXNIP knockdown promotes the VEGFR2 expression and partially restores the angiogenesis in context of NaBu treatment. (a) Western blot was used to confirm shRNA knockdown efficiency of TXNIP in HUVEC cells in context of NaBu treatment (left). Relative grey values were used for the statistical analysis (right) (^∗∗^*P* ≤ 0.01). (b) HUVEC cells stably expressing TXNIP shRNA were treated with designated concentration of NaBu, and cell lysates were subjected to western blot analysis with anti-VEGFR2 or anti-TXNIP antibody. Beta-actin was used as a loading control. Relative grey values were used for the statistical analysis (^∗∗^*P* ≤ 0.01). (c) Tube formation assay was performed with stable cell lines either expression TXNIP shRNA or scramble shRNA (control). Those two cell lines were treated with 2.5 mM NaBu, and the tube formation status was pictured (left). ImageJ was used to statistically analyzed the values of length, size, and junction of tube formation of HUVEC cells (^∗^*P* ≤ 0.05, bar: 400 *μ*m) (right). (d) MTT assay was carried out to evaluate the effect of NaBu on stable HUVEC cell lines proliferation with either TXNIP knockdown or scramble control (*n* = 5‐8, ^∗^*P* ≤ 0.05). SHNC: scramble shRNA control; TXNIP-SH4: TXNIP shRNA.

TXNIP overexpression could inhibit HUVEC cell proliferation and tube formation. We therefore decreased the TXNIP expression by small interfering RNAs (shRNAs). We used previously constructed TXNIP shRNA expression plasmids [11]. Because the shRNA TXNIP-SH4 showed the highest knockdown efficiency in A549 cells, we also used the plasmids and the corresponding scramble shRNA here. In order to test the knockdown efficiency, we constructed stable cell lines expressing TXNIP-SH4 or scramble RNA control (SHNC) with above two plasmids. The TXNIP expression in our HUVEC cell line was very low. We thus stimulated those two stable cell lines with 2.5 mM NaBu for 24 hours and then checked the TXNIP expression with western blot (Figure 5(a)). Statistically, the TXNIP protein in TXNIP-SH4 expression cells decreased to 0.71 folds comparing to the cells expressing scramble shRNA (SHNC), and *P* value is less than 0.05, indicating a significant reduction in TXNIP protein (Figure 5(a)). We also tested the VEGFR2 protein in those two cell types undergoing NaBu treatment. Surprisingly, TXNIP knockdown could significantly upregulated the VEGFR2 expression (1.46 folds) (Figure 5(b)). We further tested the ability of tube formation for those two cell types in condition of NaBu treatment. We discovered the total length and junction of tube formation increased significantly, but the tube formation size had no significant change although an obviously increment was observed (Figure 5(c)). Knockdown TXNIP also enhanced HUVEC proliferation ability in a time dependent manner (1.56 and 2.23 fold at 48 h and 72 h, respectively, *P* ≤ 0.05, Figure 5(d)). Those results further confirmed the roles of TXNIP in NaBu-mediated neovascularization suppression. Moreover, the TXNIP/VEGFR2 pathway might be a potential pathway involving in the NaBu suppressive neovascularization.

## 4. Discussion

Neovascularization (CNV) is very common in numerous diseases. Current therapeutic methods on CNV exhibit various defects. As an endogenic short chain small molecule, NaBu exhibits various physiological roles, antitumor effect, inflammation, and glucose regulation [12, 19, 36–38]. Here, we studied the effect of NaBu to the CNV in ocular tissue and established the molecular link between NaBu and TXNIP mediated inhibition of CNV. We found NaBu inhibited CNV partially through regulating the level of TNXIP, which further target the VEGFR2, an important gene used for the stimulation of vessel growth [39].

Previous study primarily confirmed that NaBu inhibited the migration and tube formation of HUVEC cells through upregulating the expression of HIF-alpha [40]. Here, we further showed NaBu inhibited the ocular angiogenesis. Especially, NaBu suppressed choroid sprouting in vitro (Figure 2) and the laser-induced CNV. Moreover, removing NaBu at the second day, the inhibited choroid continues to resprout at the next culture time (Figure 2). Currently, we cannot exclude the possibility that longer incubation time with NaBu will lead to the apoptosis of choroid tissue cells.

Those data revealed that NaBu inhibited choroid sprouting through apoptosis-independent manner. This conclusion was further supported by the TUNEL assay (data not showed).

Actually, NaBu induces a variety of genes expression. Previously, we found TXNIP was highly induced in a non-small-cell lung cancer cell line A549 [22]. Moreover, high level TXNIP often leads to cell death via inducing diverse cellular events [41–43]. In this study, we also observed a great enhancement in both mRNA and protein of TXNIP (Figure 3). Those data prompt us to examine the role of TXNIP in NaBu-mediated CNV suppression. As expected, TXNIP was found to be highly upregulated in NaBu-treated CNV (Figure 3). However, the expression and localization of TXNIP proteins on laser-induced CNV lesion were specificity. The expression of TXNIP protein was stronger in the peripheral area of the CNV lesion than its center area, where the Isolectin IB4 staining showed higher signals (Figure 3). New blood vessel formation starts with endothelial tip cell selection and vascular sprout migration, followed by the establishment of functional, perfused blood vessels [7, 44]. At the center area of CNV lesion is the endothelial cells consisting of the mature blood vessels; however, tufts rounding the center area of CNV lesion consist of tip cells. We therefore deduced that the TXNIP expression in the tip cells is more sensitive to the NaBu stimulation.

TXNIP is a regulator of redox homeostasis, glucose-induced stress, and inflammatory activity. It mediates tumorigenesis and neurodegenerative diseases [36, 45] and the pathogenesis of oxygen-induced retinal neovascularization [23, 24, 46]. As a negative regulator of transcription, TXNIP together with HDAC1/2 also involves in the expression regulation of many genes [24, 46–48]. At normal condition, TXNIP knockdown significantly decreased phosphorylation of VEGFR2 and inhibited VEGF-induced endothelial cell tube formation and proliferation [39]. In condition of NaBu treatment, TXNIP knockdown promoted tube formation, and proliferation of HUVEC cells activated the mRNA and protein expression of VEGFR2, compared to its negative control (Figure 5). Those results did not contradict with the previous reports [39]. As we noted above, NaBu actually could remodel the gene expression profile.

**Figure 6:**
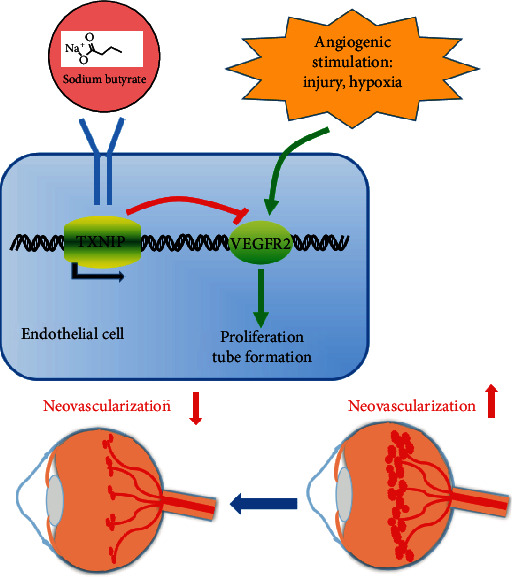
Schematic diagram illustrates the possible mechanisms on how NaBu inhibits angiogenesis. Under angiogenic stimuli such as laser and injury, the VEGFR2 expression was activated and then initiated the angiogenic activities. When treated with NaBu, the TXNIP expression in endothelial cells was upregulated and enhanced TXNIP expression negatively regulated VEGFR2 expression. As a result, the angiogenesis was prevented.

In summary, our study confirmed that NaBu effectively inhibited *in vitro* and *in vivo* angiogenesis, and this inhibitory effect partially attributed to the activation of TXNIP expression, which then regulated the VEGFR2 expression, in response to NaBu treatment (Figure 6). NaBu treatment inhibits vascular endothelial cell growth with low toxicity effect on cells and tissues; it could serve as a new candidate for the antiangiogenic treatments for neovascular ocular diseases.

Taken together, we established the molecular link between NaBu and TXNIP-regulated angiogenesis. And further found that TXNIP could regulate the VEGFR2 expression in context of NaBu treatment.

## Figures and Tables

**Table 1:** Primers and sequencing.

Gene	Origin	Forward (5′—3′)	Reverse (5′—3′)
*Actb*	Mouse	CACTGTCGAGTCGCGTCC	TCATCCATGGCGAACTGGTG
*Txnip*	Mouse	GAAGGCTTTTCTCGATCGCC	GGCAGACACTGGTGCCATTA
*Vegfr2*	Mouse	TTGTGAATGTCCCACCCCAG	TTGGCGTAGACTGTGCATGT
*ACTB*	Human	CTTCGCGGGCGACGAT	CACATAGGAATCCTTCTGACCC
*TXNIP*	Human	CGGGTGATAGTGGAGGTGTG	TTCTCACCTGTTGGCTGGTC
*NLRP3*	Human	CTAGCTGTTCCTGAGGCTGG	GTCCTTAGGCTTCGGTCCAC
*GLUT1*	Human	TGGCATCAACGCTGTCTTCT	AGCCAATGGTGGCATACACA
*CASPASE3*	Human	GGCGCTCTGGTTTTCGTTAAT	TCCAGAGTCCATTGATTCGCT

## Data Availability

The datasets used and analyzed during the current study are available from the corresponding author on reasonable request.

## Abbreviations

NaBu: Sodium butyrate
HUVEC: Human umbilical vein endothelial cell
TXNIP: Thioredoxin-interacting protein
VEGF: Vascular endothelial growth factor
VEGFR2: Vascular endothelial growth factor receptor 2
CNV: Choroidal neovascularization
NT: No transfection
E.V: Empty vector
TXNIP-OV: TXNIP overexpression
TXNIP-SH4: TXNIP SH4 knockdown.

## Supplementary Materials

Supplementary Materials Supplementary Figure 1: TXNIP expression was higher in NaBu-treated group than that of saline-treated control group. Supplementary Figure 2: NaBu showed little damage to the normal tissues confirmed by TUNEL analysis. Supplementary Figure 3: the infection efficiency of TXNIP overexpression with lentivirus plasmids and shRNA expression plasmids (stable cell line).
