# Supplementary material for: Sodium Butyrate Inhibits Neovascularization Partially via TNXIP/VEGFR2 Pathway
Source: Oxid Med Cell Longev. 2020 Nov 20;2020:6415671. doi: 10.1155/2020/6415671 (PMC7700023; doi:10.1155/2020/6415671)
Supplement: Supplementary Materials — Supplementary Figure 1: TXNIP expression was higher in NaBu-treated group than that of saline-treated control group. Supplementary Figure 2: NaBu showed little damage to the normal tissues confirmed by TUNEL analysis. Supplementary Figure 3: the infection efficiency of TXNIP overexpression with lentivirus plasmids and shRNA expression plasmids (stable cell line). [file 6415671.f1.pdf]

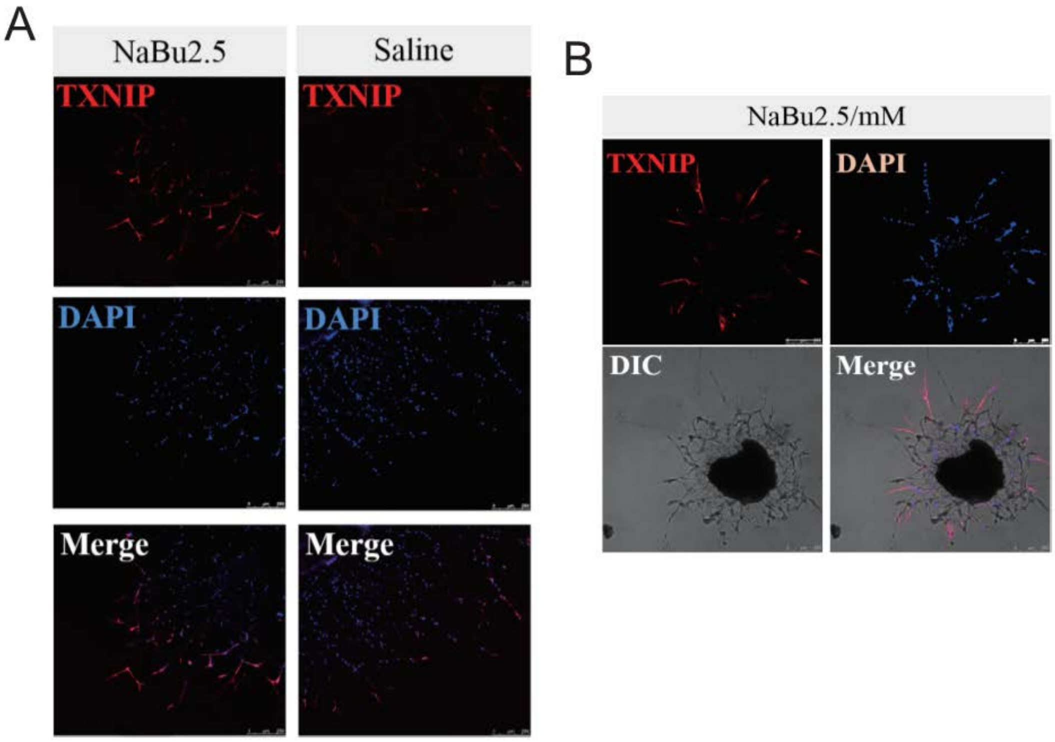

Supplementary Figure.1 TXNIP expression was higher in NaBu treated group than that of saline treated control group.

Samples choroid sprouting analysis were used for the ICC staining with TXNIP antibody , and DAPI was used for the staining of nuclei. A, left is NaBu treated group and right is the saline treated group. B, a whole choroid sprouting sample was showed after staining with TXNIP antibody. TXNIP,Red;DAPI,blue.

A

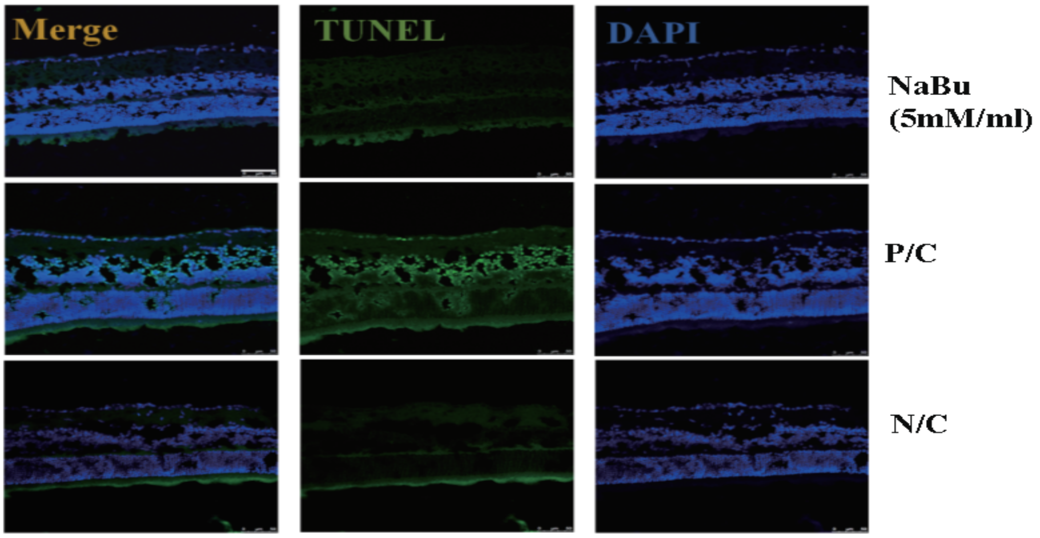

B

Sp	Av.Ap.Cm	STDEV
P/C	244	12.7279
NaBu	0.39	0.583
N/C	0.33	0.5774

Supplementary Figure.2 NaBu showed little damage to the normal tissues confirmed by TUNEL analysis.

A, Represented pictures showed the TUNEL staining in NaBu treated retina,positive control (P/C)and negative control(N/C),TUNEL ,green, DAPI (blue) staining the cellular nuclei. B, Statistical analysis for A.Av,Ap,Cm,represent the positive TUNEL staining cell number,STDEV,represent standard deviation.

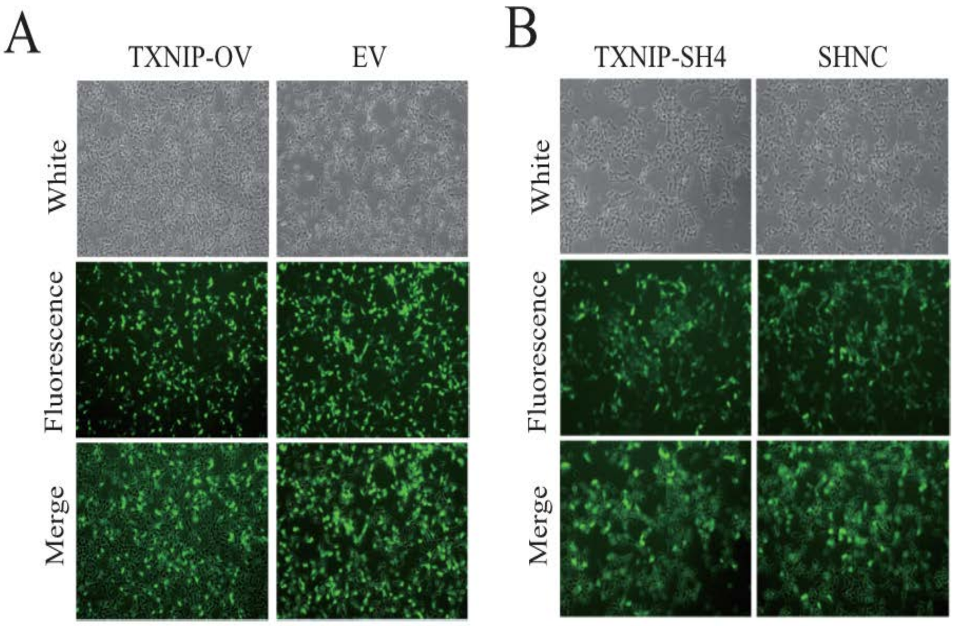

Supplementary Figure.3 the infection efficiency of TXNIP overexpression with lentivirus plasmids and shRNA expression plasmids(stable cell line).A is TXNIP overexpression,B is shRNA expression plasmids.
